# Prevalence of pilus islets and association with clonal complex in *Streptococcus pneumoniae* isolated from children in Suzhou, China

**DOI:** 10.1128/spectrum.02529-24

**Published:** 2025-03-31

**Authors:** Jiaming Shen, Xiaofei Liu, Lili Huang, Youyi Zhang, Yunzhen Tao, Mengzhen Wang, Genming Zhao, Xuejun Shao, Tao Zhang

**Affiliations:** 1 School of Public Health, Fudan University, Key Laboratory of Public Health Safety, Ministry of Education 70581 , Shanghai, China; 2 Zhangjiang Community Health Service Center, Shanghai, China; 3 Children’s Hospital of Soochow University 12582, Suzhou, Jiangsu, China; MultiCare Health System, Tacoma, Washington, USA

**Keywords:** *Streptococcus pneumoniae*, pilus islets, clonal complex, children, antibiotic susceptibility

## Abstract

Pilus islets (PIs) in *Streptococcus pneumoniae* play a crucial role in bacterial adhesion and virulence. This study aims to investigate the prevalence of PIs and explore their associations with serotypes, clones, and antibiotic susceptibility in *S. pneumoniae* isolated from children in Suzhou, China. A total of 341 *S*. *pneumoniae* isolates from aseptic specimens, ear secretions, and sputum between 2018 and 2021 were analyzed. Serotyping was conducted using latex and Quellung reactions. Antimicrobial susceptibility testing was performed using E-test methods. Multi-locus sequence typing (MLST) was conducted to identify the sequence types and clonal complex (CC). PI-1 and PI-2 were detected by PCR assays for the *rlr*A and *sip*A genes, respectively. A total of 63.6% of the included *S. pneumoniae* isolates expressed at least one type of PI, with strains of PCV13 vaccine serotypes showing a significantly higher positive rate of PIs than non-vaccine serotype strains (71.6% vs. 24.5%). PI prevalence varied across sample sources and clonal complexes. While a stronger association of PIs with clones than with serotypes was identified, most of the piliated isolates belonged to prevalent CCs, such as CC271. In addition, piliated isolates tended to be resistant to antibiotics compared to the non-piliated isolates. Our study highlights the high prevalence of PIs and the implications on antibiotic resistance and clonal dissemination of *S. pneumoniae* among children in Suzhou, China.

**IMPORTANCE:**

This study reveals a high prevalence of PIs in *Streptococcus pneumoniae* among children in China, which differs from isolates in other countries and highlights their implications for antibiotic resistance and clonal dissemination. To our knowledge, we are the first to report PI prevalence across different clinical samples within the same population, suggesting a potential link between PIs and acute otitis media (AOM) in Chinese children. These findings contribute valuable insights into the role of PIs in clinical settings and underline the need for targeted interventions, including vaccine strategies and antimicrobial stewardship protocols. By advancing our knowledge of PI epidemiology, this research enriches the existing literature and aims to inform public health initiatives, ultimately improving health outcomes for vulnerable pediatric populations.

## INTRODUCTION

Pneumococcal disease (PD) is a serious public health concern that encompasses a range of illnesses, ranging from acute otitis media to life-threatening invasive diseases, such as bacteremia and meningitis. The World Health Organization (WHO) reports that PD is responsible for approximately 1.6 million deaths annually, with over 60% occurring in children < 5 years of age and mostly in developing countries (1). Given the severity of this disease, the WHO has classified PD as requiring “extremely high priority” for vaccine prevention (2). Immunization with pneumococcal conjugate vaccines (PCVs) has been considered the most effective measure to control pneumococcal infections. While PCVs target only a limited number of serotypes, serotype replacement has been observed across all regions widely using PCVs (3). However, the development of higher-valency vaccines faces obstacles, and the emergence of non-vaccine serotypes following PCV use underscores the need for some universal vaccine targets.

Pilus islets (PIs), including pilus islet 1 (PI-1) and pilus islet 2 (PI-2), are long proteinaceous, polymeric structures anchored on the surface of *S. pneumoniae* (4). PIs play a crucial role in pneumococcal adhesion and virulence by facilitating bacterial attachment to host cells (5). In a recent study, a fusion protein, called RrgB321, consisting of three pilus-1 backbone *rrg*B variants, was found to successfully protect mice against both high- and low-level pilus-expressing strains of *S. pneumoniae* (6). Additionally, PI protein subunits have shown promise as immunogenic epitopes in murine infection models, generating protective antibodies (7). Thus, PI, as a potential vaccine candidate, has resulted in growing interest.

The reported prevalence of PI-1 and PI-2 ranges from 10% to 35%, with a higher rate observed in antibiotic-resistant *S. pneumoniae* isolates (8). However, one recent study in China reported significantly elevated prevalence rates, with PI-1 and PI-2 rates of 59.5% and 67.6%, respectively (9). Moreover, antibiotic resistance rates among pneumococcal isolates in China were notably high, with 94.4% resistance to erythromycin, 92.3% to clindamycin, and 32.0% to penicillin (10). Considering such a high prevalence of antibiotic-resistant pneumococcal isolates, PIs may play a key role in the dissemination of antibiotic-resistant pneumococcal strains in China. However, due to limited evidence, it is still a challenge to draw any conclusion. Further investigations on the association of PIs and antibiotic susceptibility are needed.

Given the urgency to curb the spread of antibiotic-resistant *S. pneumoniae* isolates and mitigate the impact of PD, gaining knowledge about the prevalence and driving force of endemic piliated pneumococci is crucial. Therefore, with the prospectively collected clinical *S. pneumoniae* strains isolated from hospitalized children in Suzhou, China, we tried to investigate the prevalence of PI-1 and PI-2 and evaluate potential associations between PIs and serotypes, antibiotic susceptibility, and clones.

## MATERIALS AND METHODS

### Pneumococcal isolates and serotyping

From 2018 to 2021, a prospective collection of clinical *S. pneumoniae* isolates was carried out at the Children’s Hospital of Soochow University (SCH) in Jiangsu Province, China (11). A total of 341 *S*. *pneumoniae* isolates were included in this study, including all isolates collected from aseptic specimens (*n* = 38) and ear secretions (*n* = 151). For isolates collected from sputum (n=5874), we employed simple random sampling to select 152 isolates for inclusion. This study has been approved by the Ethics Committee of Fudan University School of Public Health (IRB#2017–11-0646).

Strains isolated from aseptic specimens (e.g., cerebrospinal fluid, blood, and pleural fluid) were defined as invasive pneumococcal disease (IPD) strains, while others were defined as noninvasive pneumococcal disease (NIPD) strains. Clinical information of patients with positive pneumococcal cultures was retrospectively collected through individual chart review. Serotypes were identified via latex and Quellung reactions (Staten’s Serum Institute, Copenhagen, Denmark) (12).

### Antibiotic susceptibility tests

The susceptibility of pneumococci was determined using the E-test method by the clinical laboratory of SCH. The antimicrobial agents included erythromycin, tetracycline, clindamycin, trimethoprim-sulfamethoxazole, penicillin, amoxicillin, cefotaxime, levofloxacin, moxifloxacin, quinupristin/dalfopristin, chloramphenicol, rifampin, vancomycin, and linezolid. The decisions concerning the necessity of antibiotic susceptibility testing and the selection of specific antibiotics to be tested were made by clinical doctors, taking into account the treatment requirements. Quality control analysis was performed using *S. pneumoniae* ATCC49619. The results were interpreted according to the latest Clinical and Laboratory Standards Institute (CLSI) standard (13). Multidrug resistance (MDR) is defined as resistance to at least three different types of antibiotics, while extensive drug resistance (XDR) is defined as resistance to at least five different types of antibiotics.

### Multi-locus sequence typing (MLST)

Internal fragments of seven housekeeping genes (*aroE*, *gdh*, *gki*, *recP*, *spi*, *xpt*, and *ddl*) were amplified by PCR and sequenced (14). The following thermocycling conditions were used: 5-minute hold at 94°C, followed by 30 cycles of 94°C for 15 seconds, 54°C for 30 seconds, and 72°C for 45 seconds, and a final extension at 72°C for 10 minutes. Sequences were submitted to the MLST database (http://pubmlst.org/spneumoniae/) to identify allelic profiles and sequence types (STs).

### Detection of Pilus Islets

PI-1 and PI-2 were detected by PCR for the *rlr*A (15) and *sip*A genes, respectively (Table S1). The *cps*A (12) gene was used as a species-specific positive control. The following thermocycling conditions were used: 5-minute hold at 94°C, followed by 30 cycles of 94°C for 30 seconds, 55°C for 30 seconds, and 72°C for 1 minute, and a final extension at 72°C for 5 minutes. PCR products were run on 2% agarose gels at 130 V for 25 minutes. Isolates that tested positive for either the PI-1 or PI-2 gene were considered as piliated *S. pneumoniae* isolates.

### Statistical analysis

PHYLOViZ software (16) was utilized to assign isolates to clonal complexes (CCs), defined as clusters sharing at least five out of seven alleles. CCs were named after the ST of the goeBURST-predicted founder.

R4.3.0 was used to analyze the data. The Pearson χ test was used to compare the proportions. The changes in the prevalence of PI presence over the years were assessed using the Cochran-Armitage test for trends. The differences were considered statistically significant when *P* < 0.05.

The index of discrimination (IOD) was calculated as IOD = 1 − ∑ [*n*_*j*_ (*n*_*j*_ − 1)]/[*N*(*N* − 1)], where *n*_*j*_ is the number of isolates belonging to the *j*th pattern and *N* is the number of total isolates (17). The value of IOD ranges from 0 to 1, with higher values representing a higher degree of diversity of sequence types in the population.

## RESULTS

### Characteristics of the included *S. pneumoniae* isolates

Out of the 341 strains, 88.3% (*n* = 301) were isolated from children under five years old (Table 1). Among the IPD cases, 71.1% (27/38) were bacteremia, 23.7% (9/38) were meningitis, and 5.3% (2/38) were pleural and peritoneal inflammation. The most prevalent NIPD diseases were pneumonia (48.5%, 147/303) and otitis media (49.8%, 151/303). Other conditions included asthma, pulmonary hypertension, and foreign body in the airway.

**TABLE 1:** Basic information of the included isolates and patients during 2018–2021

	Sputum (*N* = 152) *N* (%)	Ear secretion (*N* = 151) *N* (%)	Aseptic specimen (*N* = 38) *N* (%)
Isolation information			
Isolated year			
2018	41 (27.0)	58 (38.4)	9 (23.7)
2019	48 (31.6)	49 (32.5)	8 (21.1)
2020	39 (25.7)	24 (15.9)	10 (26.3)
2021	24 (15.8)	20 (13.2)	11 (28.9)
Isolated season			
Spring	33 (21.7)	33 (21.9)	11 (28.9)
Summer	25 (16.4)	15 (9.9)	6 (15.8)
Autumn	52 (34.2)	50 (33.1)	10 (26.3)
Winter	42 (27.6)	53 (35.1)	11 (28.9)
Antibiotic susceptibility tests			
Yes	134 (88.2)	124 (82.1)	35 (92.1)
No	18 (11.8)	27 (17.9)	3 (7.9)
Patient demographics			
Sex			
Male	92 (60.5)	92 (60.9)	27 (71.1)
Female	60 (39.5)	59 (39.1)	11 (28.9)
Age (yr)			
<1	35 (23.0)	43 (28.5)	4 (10.5)
1–	35 (23.0)	24 (15.9)	7 (18.4)
2–	58 (38.2)	74 (49.0)	21 (55.3)
≥5	24 (15.8)	10 (6.6)	6 (15.8)

**Fig 1:**
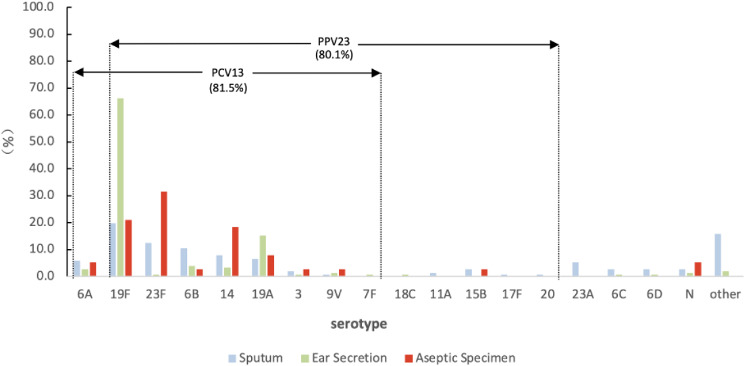
Serotype distribution of pneumococcal strains isolated from different source specimens. “*N*” denotes non-typeable; “other” includes serotypes 9C, 11B, 11C, 13, 15A, 15C, 15F, 19B, 19C, 21, 23B, 24, 33D, 35A, 35B, 35F, 36, 39, and 48; each of the above serotypes occurred less than three times.

A total of 37 serotypes were identified, with 7 isolates being non-typeable. The PCV13 vaccine serotype coverage was 81.5%. Among strains isolated from aseptic specimens, the most prevalent serotypes were 23F (31.6%, 12/38), 19F (21.1%, 8/38), and 14 (18.4%, 7/38), accounting for 71.1% of cases. The dominant serotypes in ear secretions were 19F (66.2%, 100/151) and 19A (15.2%, 23/151), accounting for 81.4% of cases. A wide variety of serotypes were observed in the strains obtained from sputum, among which 19F, 23F, 6B, 14, and 19A were the top five serotypes (Fig. 1).

### Prevalence of pilus islets

Of the 341 isolates, 217 (63.6%) expressed at least one PI, and 112 (32.8%) carried both PIs. Specifically, 57.5% (196/341) of the isolates were positive for PI-1, and 39.0% (133/341) were positive for PI-2. The positive rate of PI-1 was significantly higher than that of PI-2 (χ^2^ = 23.307, *P* < 0.001). PI prevalence was highest in isolates from ear secretions, followed by sputum, with the lowest prevalence in aseptic specimens (Table 2). Among the isolates expressing PI-2, the majority (84.2%, 112/133) expressed PI-1 concurrently. The proportion of piliated isolates decreased from 71.3% in 2018 to 49.3% in 2020 (*P* = 0.004) but rebounded in 2021 (61.8%). (Fig. S1)

**TABLE 2:** Prevalence of pilus islets in isolates from different sources^*a*^

	Overall (*N* = 341)	Sources		
		Sputum (*N* = 152)	Ear secretion (*N* = 151)	Aseptic specimen (*N* = 38)
PIs prevalence				
Piliated	217 (63.6)	69 (45.4)	129 (85.4)	15 (39.5)
PI-1+	196 (57.5)	64 (42.1)	118 (78.1)	12 (31.6)
PI-2+	133 (39.0)	51 (33.6)	69 (45.7)	10 (26.3)
Strain distribution				
PI-1+ only	84 (24.6)	18 (11.8)	60 (39.7)	5 (13.2)
PI-2+ only	21 (6.2)	5 (3.3)	11 (7.3)	3 (7.9)
Both PI-1+ and PI-2+	112 (32.8)	46 (30.3)	58 (38.4)	7 (18.4)
Neither	124 (36.4)	83 (54.6)	22 (14.6)	23 (60.5)

^*a*^ “Piliated” refers to all isolates that tested positive for either the PI-1 or PI-2 gene. This includes the sum of strains in the “PI-1+ only,” “PI-2+ only,” and “both PI-1+ and PI-2+” categories in the “Strain distribution” section. “PI-1+” refers to all isolates that tested positive for the PI-1 gene, encompassing the sum of strains in both the “PI-1+ only” and “both PI-1+ and PI-2+” categories in “Strain distribution.” Similarly, “PI-2+” refers to all isolates that tested positive for the PI-2 gene, which includes the sum of strains in both the “PI-2+ only” and “both PI-1+ and PI-2+” categories in “Strain distribution.”

### Association of the pilus islets with serotypes

**Fig 2:**
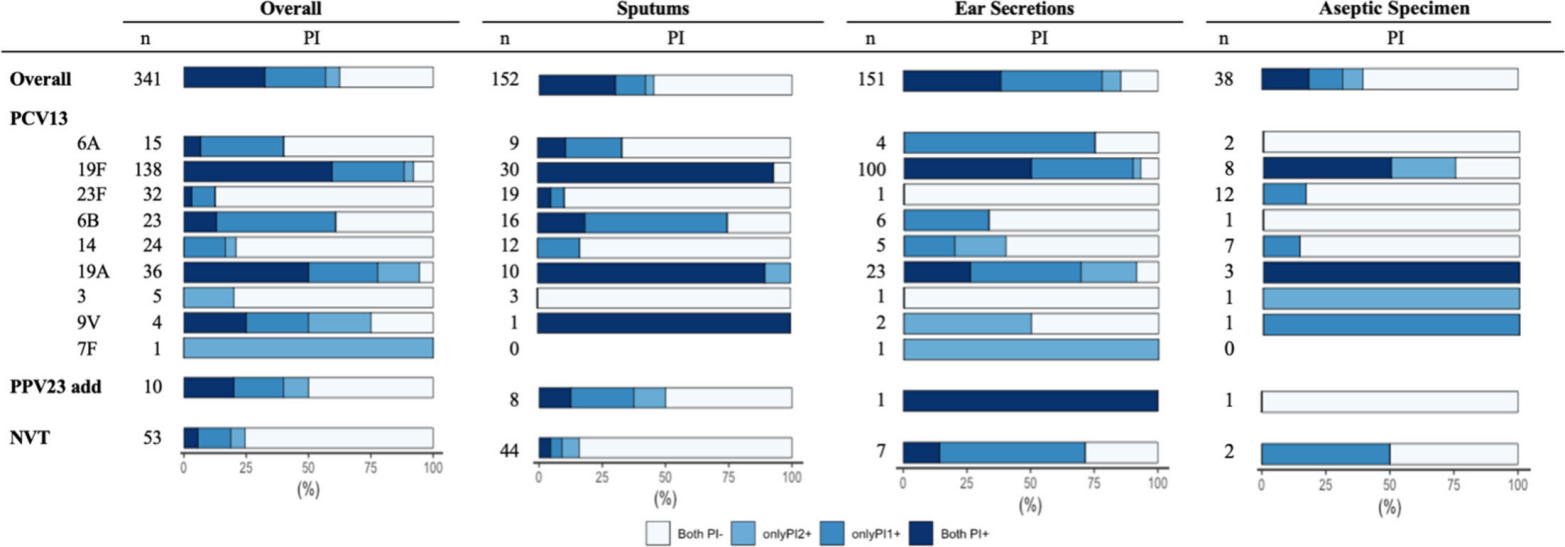
Distribution of pilus islets in *S. pneumoniae* isolates with different serotypes.

As shown in Fig. 2, the prevalence of PIs differed by serotypes. Isolates of PCV13 vaccine serotypes (VT) were far more likely to have PIs than non-vaccine type (NVT) isolates (frequencies of piliated pneumococci: VT vs. NVT: 71.6% vs. 24.5%; *P* < 0.001). In particular, serotypes 19F, 19A, and 7F showed exceptionally high rates of PI

presence, exceeding 90%. Serotypes 23F, 14, and 3 exhibited a relatively low prevalence of PI presence. Although isolates from diverse sources exhibited varying degrees of PI prevalence, isolates belonging to the same serotype showed a similar PI prevalence across different sources.

### Association of pilus islets and antibiotic susceptibility

A total of 85.9% (293/341) of the isolates were tested for antibiotic susceptibility, and all of them were resistant to erythromycin but susceptible to vancomycin, levofloxacin, and linezolid. With the exception of quinupristin/dalfopristin, piliated pneumococci exhibited higher resistance to several antibiotics than non-piliated pneumococci, such as tetracycline, trimethoprim-sulfamethoxazole, amoxicillin, cefotaxime, and penicillin (*P* < 0.05) (Table 3). Moreover, the prevalence of MDR and XDR strains was higher in piliated isolates than in non-piliated isolates (MDR: 85.0% vs. 80.5%, χ^2^ = 0.87, *P* = 0.352; XDR: 57.7% vs. 43.0%, χ^2^ = 6.42, *P* = 0.011).

**TABLE 3:** Differences in antibiotic susceptibility among piliated (PIs+) and non-piliated (PIs−) *S. pneumoniae* isolates^*a*^

Antibiotic	PIs+		PIs−		χ^2^	*P*
	Test (*N*)	NS (%)	Test (*N*)	NS (%)		
ERY	188	100.0	105	100.0	–	–
CLI	172	99.4	99	97.0	1.18	0.277
TCY	188	95.2	105	86.7	6.80	0.009
SXT	188	96.8	105	71.4	40.27	<0.001
QDA	172	72.7	99	80.8	2.26	0.133
AMX	172	61.0	97	2.1	90.08	<0.001
CTX	188	55.3	105	4.8	73.71	<0.001
PEN	188	23.4	105	6.7	12.13	<0.001
CHL	188	5.3	105	2.9	0.47	0.493
RIF	172	0.0	99	1.0	0.08	0.779
VAN	188	0.0	105	0.0	–	–
LVX	188	0.0	105	0.0	–	–
LNZ	188	0.0	105	0.0	–	–

^*a*^ NS (non-susceptible) includes both resistant (R) and intermediate (I) strains. The dash (–) indicates that statistical analysis (χ² test) was not performed or applicable. Antibiotics include erythromycin (ERY), tetracycline (TCY), clindamycin (CLI), tetracycline (TET), trimethoprim-sulfamethoxazole (SXT), penicillin (PEN), amoxicillin (AMX), cefotaxime (CTX), levofloxacin (LVX), moxifloxacin (MXF), quinupristin/dalfopristin (QDA), chloramphenicol (CHL), rifampin (RFP), vancomycin (VAN), and linezolid (LZD). A total of 293 strains were tested for antibiotic susceptibility, but not all of them were tested for every type of antibiotic, resulting in varying test numbers for each antibiotic.

### Pilus islet presence by clonal complex

**Fig 3:**
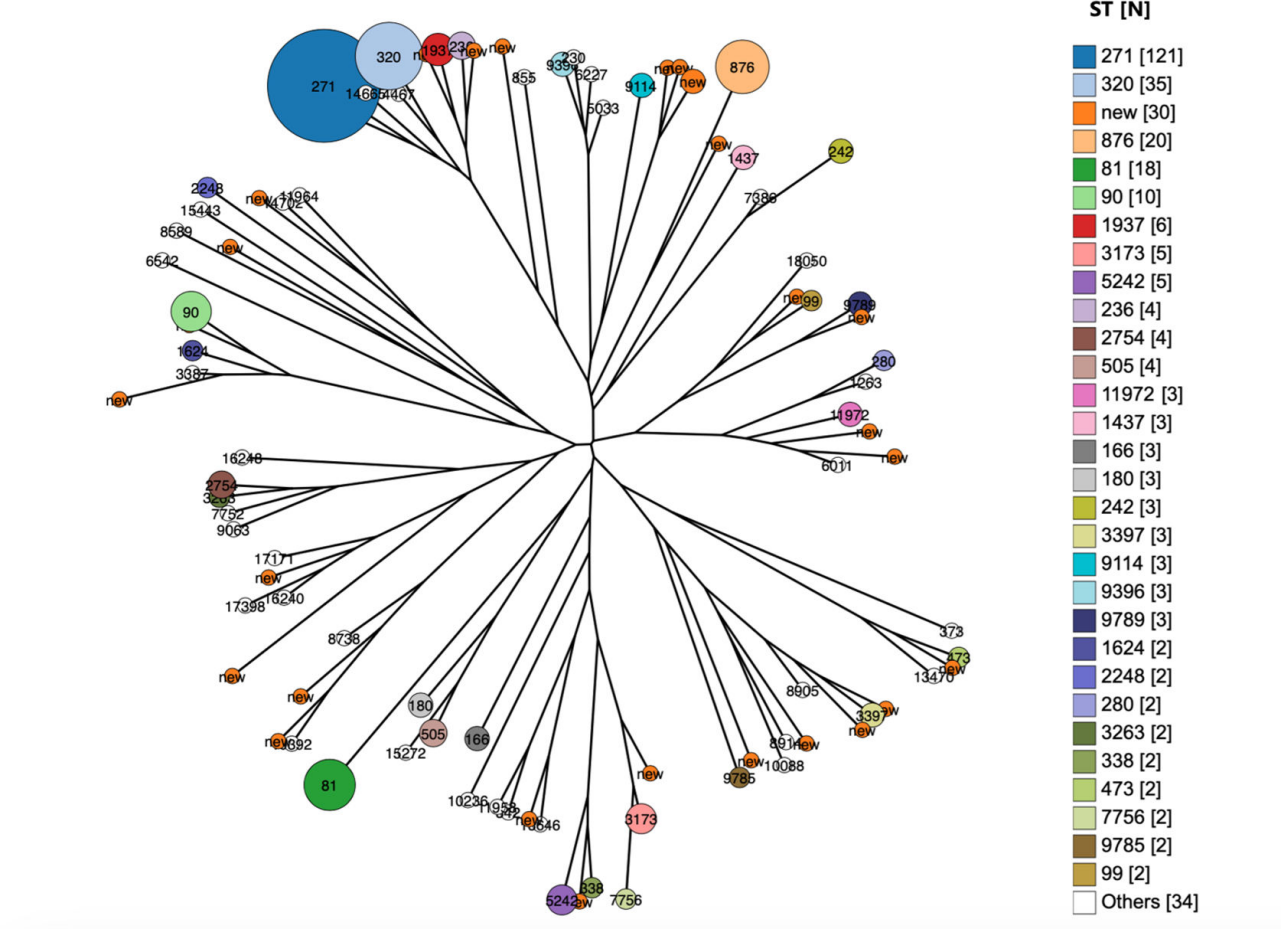
Population snapshot of 341 *S*. *pneumoniae* isolates in this study. One spot represents a single ST. The size of the circle corresponds to the number of isolates belonging to a ST. The lines indicate the presence of single locus variant links among particular STs by neighbor-joining.

Among the 341 isolates, 88 STs were identified, with 63 known STs and 25 new STs identified in 30 isolates (Fig. 3). Isolates from aseptic specimens (IOD = 0.923) and sputum (IOD = 0.929) sources had higher levels of diversity than those from ear secretions (IOD = 0.661). However, there was no significant diversity between non-piliated isolates from the three sources (*P* > 0.05). Piliated isolates, especially those carrying both PIs, have lower levels of diversity than non-piliated *S. pneumoniae* isolates (Table S2).

**Fig 4:**
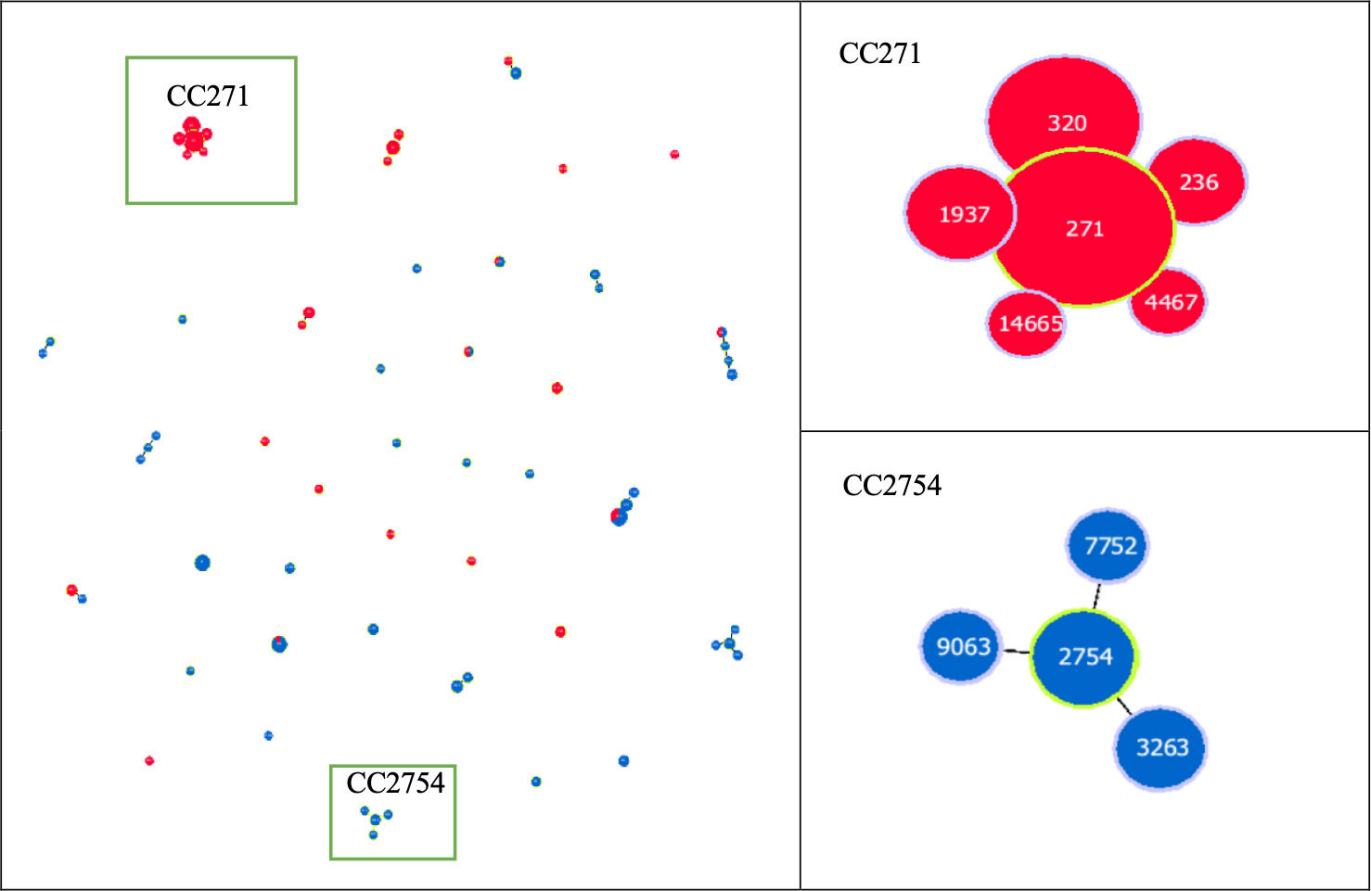
Minimum spanning tree analysis of 341 *S*. *pneumoniae* isolates in this study. Categorical clustering was performed based on MLST. In the minimum spanning tree, the STs are displayed as circles. The size of each circle indicates the number of isolates within this particular type. Relationships between the STs are depicted through the lines connecting the STs and the relative lengths of the branches linking them. Piliated *S. pneumoniae* isolates and non-piliated *S. pneumoniae* isolates are represented by red and blue, respectively.

According to the MLST and goeBURST analyses, 15 CCs and 17 singletons were identified (Fig. 4). CC271 (*n*  =  168, 49.3%), mainly consisting of Taiwan19F-14, was the most predominant CC.

A strong association between PI presence and CCs was observed. The prevalence of PIs showed a high consistency across most CCs. For example, all isolates in CC271 were piliated, whereas none in CC2754 carried PIs, including one strain of serotype 19F. Additionally, except for strains in CC81 (Spain^23F^-1), all other isolates in CCs recognized by the Pneumococcal Molecular Epidemiology Network (PMEN) (18) carried PIs (Table S3).

## DISCUSSION

In this study, we investigated the prevalence of PIs and their association with specimen sources, serotypes, antibiotic resistance, and clonal complexes in pneumococcal isolates from China. The findings revealed that 63.6% of the *S. pneumoniae* isolates expressed at least one type of PI. The prevalence of PI-1 (57.5%) was notably higher than previously reported rates (10%–35%) in other studies (19–21), while PI-2 (39.0%) aligned with prior studies from China (19) but exceeded the rates (16%–21%) reported elsewhere (5, 22).

The high PI prevalence observed in our study warrants further investigation. First, the isolates from ear secretions exhibited the highest PI prevalence (PI-1: 78.1%, PI-2: 45.7%) among the three types of resources, contributing to the overall high prevalence observed. The high prevalence of piliated pneumococci from ear secretions also suggests a potential clinical relevance between the PIs and AOM in Chinese children. Furthermore, our analysis of serotypes revealed a higher prevalence of PIs in PCV13 serotypes than in non-vaccine types (NVTs), consistent with findings from Portugal, where 83% of piliated pneumococcal isolates were covered by PCV7 serotypes (4, 6B, 9V, 14, and 19F) (15). In countries where PCVs are included in national immunization programs (NIP), the prevalence of piliated pneumococci has declined significantly following the vaccine introduction (23, 24). However, in China, where PCV13 serotypes still dominate in clinical isolates and the PCV vaccination rate is low due to the self-funded immunization strategy (25, 26), the lack of vaccine impact likely contributed to the high prevalence of PIs in our study.

Our findings provide important insights into the relationship between PIs and CCs. Piliated isolates exhibited lower levels of clonal diversity than non-piliated *S. pneumoniae* isolates, suggesting that they often belong to specific CCs. As described in previous studies, the presence of PIs is a clonal property (15). In this study, the differences in PI prevalence rate across different CCs, as well as the high consistency of PI presence within the same CC, further support this perspective. However, such consistency was not as evident across serotypes. Notably, a majority of piliated *S. pneumoniae* strains belong to specific dominant CCs, such as CC271. The role of PIs in promoting colonization and enhancing epithelial adhesion is supported by compelling evidence from laboratory mouse experiments (27). It is plausible that piliated isolates could have an advantage in colonization, thus facilitating the widespread dissemination of these prevalent lineages within the population. Moreover, we observed that despite belonging to the same serotype, different clones exhibited varying prevalences of PIs. For example, although over 90% of serotype 19F isolates express PIs, isolates from CCs, such as CC9396 (ST6227), CC11958 (ST13646), and CC2754 (ST2754), which consist entirely of non-piliated isolates, do not express PIs. These observations indicate that the association between PIs and clones is stronger than that with serotypes.

Additionally, with regard to antibiotic resistance, most of the isolates related to PMEN clones possess PIs. Our findings were consistent with previous reports (28, 29), highlighting that piliated pneumococci have an increased tendency to be resistant to antibiotics (21, 30). While the exact mechanisms remain unclear, one possibility is that PIs promote prolonged colonization, making these piliated pneumococci more susceptible to selective pressure from antimicrobial agents (31). This increased exposure can potentially contribute to the acquisition of antibiotic resistance traits.

However, we acknowledge several limitations of our study. First, our strains were collected solely from one hospital, and the sample size was limited, which may have impacted their representativeness. Nevertheless, the characteristics and serotype distribution of our study isolates were similar to those observed in prospective studies in China (32, 33), providing some reassurance. Second, the duration of inclusion was not long enough to be sufficient to analyze the long-term changes in the prevalence of PIs. Third, we used the E-test method to determine pneumococcal susceptibility, which may underestimate the penicillin MIC value, leading to an inaccurate assessment of resistance rates. Lastly, our study did not delve deeply into the functional aspects of PI. Further studies are needed to evaluate the underlying mechanisms of PI virulence and pathogenesis, as well as ensure the continued surveillance of their prevalence.

### Conclusions

Our study provides evidence of the prevalence of PIs and emphasizes the critical role that PIs play in the antibiotic resistance patterns and clonal dissemination of *S. pneumoniae*. The high prevalence of PIs in China suggests their potential value as an addition to a multivalent pneumococcal protein vaccine, which potentially mitigates the spread of antimicrobial-resistant clones and effectively reduces the likelihood of disease, particularly AOM.

## Data Availability

The data that support the findings of this study are openly available in PubMLST database at https://pubmlst.org, submission id: BIGSdb_20240118125640_267124_66705, submission date: March 8, 2024 or from the corresponding author upon request.

## SUPPLEMENTAL MATERIAL

Supplemental material spectrum.02529-24-s0001.docx Tables S1 to S3; Fig. S1.

ASM does not own the copyrights to Supplemental Material that may be linked to, or accessed through, an article. The authors have granted ASM a non-exclusive, world-wide license to publish the Supplemental Material files. Please contact the corresponding author directly for reuse.
