# Supplementary material for: Prevalence of pilus islets and association with clonal complex in Streptococcus pneumoniae isolated from children in Suzhou, China
Source: Microbiol Spectr. 2025 Mar 31;13(5):e02529-24. doi: 10.1128/spectrum.02529-24 (PMC12054040; doi:10.1128/spectrum.02529-24)
Supplement: Supplemental material — Tables S1 to S3; Fig. S1. [file spectrum.02529-24-s0001.docx]

# Appendix. Supplementary material

**Supplemental Table 1**. Primer sequences of genes for PIs detection

| Gene locus | Forward primer (5’-3’) | Reverse primer (5’-3’) | Fragment size(bp) | |
| --- | --- | --- | --- | --- |
| *rlr*A(PI-1) | TCTGATAGATGAGACGCTGTTG | CTCCGCTTCTTTCTACTACAAG | 1177 |  |
| *sip*A(PI-2) | CTCTAGGAGGGATCTTCTTTATCATC | CTACAGCCGTTGTTCGATTGTCC | 550 |  |
| *cps*A | GCAGTACAGCAGTTTGTTGGACTGACC | GAATATTTTCATTATCAGTCCCAGTC | 160 |  |

**Supplemental Table 2**. Genetic diversity based on MLST of *S. pneumoniae* isolates with different PI expression.

|  |  | **Sputum** | | | | |  | **Ear Secretion** | | | | | | |  | **Aseptic Specimen** | | | | | |
| --- | --- | --- | --- | --- | --- | --- | --- | --- | --- | --- | --- | --- | --- | --- | --- | --- | --- | --- | --- | --- | --- |
|  |  | No. of strains | | No. of STs | | IOD |  | No. of strains | | No. of STs | | IOD | |  | | No. of strains | | No. of STs | | IOD |  |
| Overall | | 152 | 46 | | 0.929 | |  | 151 | 23 | | 0.661 | |  | | | 38 | 20 | | 0.923 | |  |
| PI | |  |  | |  | |  |  |  | |  | |  | | |  |  | |  | |  |
|  | PI- | 83 | 31 | | 0.935 | |  | 22 | 13 | | 0.935 | |  | | | 23 | 14 | | 0.901 | |  |
|  | Either PI+ | 69 | 17 | | 0.781 | |  | 129 | 15 | | 0.555 | |  | | | 15 | 9 | | 0.858 | |  |
|  | PI-1+ | 64 | 13 | | 0.749 | |  | 118 | 14 | | 0.529 | |  | | | 12 | 8 | | 0.894 | |  |
|  | PI-2+ | 51 | 12 | | 0.629 | |  | 69 | 8 | | 0.541 | |  | | | 10 | 5 | | 0.667 | |  |
|  | Both PI+ | 46 | 8 | | 0.551 | |  | 58 | 7 | | 0.484 | |  | | | 7 | 4 | | 0.714 | |  |

**Supplemental Table 3**. The proportion of piliated *S. pneumoniae* isolates in each clonal complex and singletons.

| Clonal Complex | Sequence Type | Serotype（N） | Piliated(%) | |
| --- | --- | --- | --- | --- |
| CC271 | 271 | 19F(108), 19A(8), 19B(1), 19C(1), 9V(1), N(2) | 100.0 |  |
|  | 320 | 19A(24), 19F(9), 23F(1), 6A(1) | 100.0 |  |
|  | 1937 | 19F(5), 19A(1) | 100.0 |  |
|  | 236_(Taiwan19F-14)_ | 19F(4) | 100.0 |  |
|  | 4467 | 19A(1) | 100.0 |  |
|  | 14665 | 19F(1) | 100.0 |  |
| CC2754 | 2754 | 19F(1), 6B(1), 13(1), 24(1) | 0.0 |  |
|  | 3263 | 6B(2) | 0.0 |  |
|  | 7752 | 35A(1) | 0.0 |  |
|  | 9063 | 9C(1) | 0.0 |  |
| CC3397 | 3397 | 14(2), 15B(1) | 100.0 |  |
|  | 8905 | 23F(1) | 0.0 |  |
|  | 8914 | 15F(1) | 100.0 |  |
|  | 10088 | 15B(1) | 100.0 |  |
| CC9396 | 9396 | 23A(1), 23B(1), 23F(1) | 0.0 |  |
|  | 6227 | 19F(1) | 0.0 |  |
|  | 5033 | 23F(1) | 0.0 |  |
|  | 230 | 23F(1) | 0.0 |  |
| CC263 | 1263 | 3 (1) | 0.0 |  |
|  | 6011 | 15F (1) | 0.0 |  |
|  | 280 | 19A (1), 9V (1) | 50.0 |  |
|  | 11972 | 15A (2), 6A (1) | 0.0 |  |
| CC505 | 505 | 3 (2), N (2) | 0.0 |  |
|  | 15272 | 6B (1) | 100.0 |  |
|  | 180 | 3 (2), 6B (1) | 0.0 |  |
| CC3173 | 3173 | 6A (4), 6C (1) | 0.0 |  |
|  | new | 6B(5), 19F (4), 6C (4), 6A(3),15C(2),N(3), 11B(1),15A(1),15F(1),17F(1),21(1),23A(1),35F(1),6D(1),7F(1) | 33.3 |  |
|  | 7756 | 6B (1), 6D (1) | 0.0 |  |
| CC11958 | 11958 | 23F (1) | 0.0 |  |
|  | 342 | 23F (1) | 0.0 |  |
|  | 13646 | 19F (1) | 0.0 |  |
| CC16240 | 16240 | 23F (1) | 0.0 |  |
|  | 17171 | 23F (1) | 0.0 |  |
|  | 17398 | 19A (1) | 0.0 |  |
| CC90 | 90_(Spain6B-2)_ | 6B(7), 6A(1), 6D(2) | 100.0 |  |
|  | 1624 | 6B(2) | 100.0 |  |
|  | 3387 | 6B(1) | 100.0 |  |
| CC5242 | 5242 | 23A (3), 23F (2) | 0.0 |  |
|  | 338 | 23A (2) | 0.0 |  |
| CC99 | 99 | 11A (1), 11C (1) | 0.0 |  |
|  | 18050 | 11C (1) | 100.0 |  |
| CC8738 | 8738 | 6D (1) | 0.0 |  |
|  | 10392 | 6B (1) | 0.0 |  |
| CC473 | 473 | 23F (1), 6A (1) | 0.0 |  |
|  | 13470 | 6A (1) | 0.0 |  |
| CC242 | 242_(Taiwan23F-15)_ | 23F(3) | 100.0 |  |
|  | 7386 | 23F(1) | 100.0 |  |
| Singletons | 81_(Spain23F-1)_ | 23F(14), 19B(1), 19F(1), 33D(1), 39(1) | 0.0 |  |
|  | 166_(SLV of Spain9V-3)_ | 9V(2) ,11A(1) | 100.0 |  |
|  | 9789 | 6A (2), 6B (1) | 100.0 |  |
|  | 876 | 14 (19), 19F (1) | 10.0 |  |
|  | 1437 | 23F (2), 23A (1) | 0.0 |  |
|  | 11964 | 14 (1) | 0.0 |  |
|  | 6542 | 36 (1) | 100.0 |  |
|  | 15443 | 48 (1) | 0.0 |  |
|  | 9785 | 15B (2) | 0.0 |  |
|  | 9114 | 19F (2), 20 (1) | 33.3 |  |
|  | 14702 | 35F (1) | 100.0 |  |
|  | 10236 | N (1) | 0.0 |  |
|  | 16248 | 18C (1) | 100.0 |  |
|  | 2248 | 14 (2) | 50.0 |  |
|  | 373 | 35B (1) | 100.0 |  |
|  | 855 | 6A (1) | 0.0 |  |
|  | 8589 | 15B (1) | 0.0 |  |

Notes: “new” refers to novel STs that have not previously been defined in the MLST database.


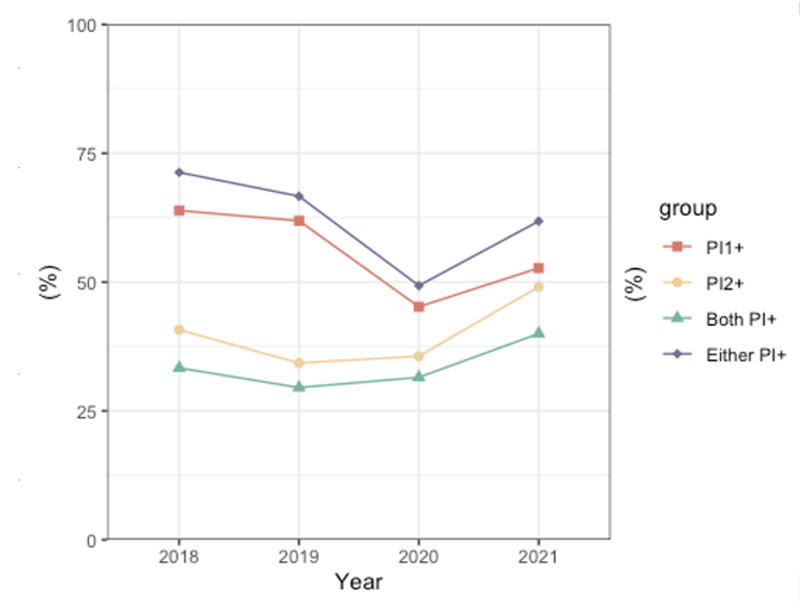


**Supplemental Figure 1**. Prevalence of Pilus Islets in isolates among different years.
